# Posttraumatic stress disorder (PTSD) and depression severity in sexually assaulted women: hypothalamic-pituitary-adrenal (HPA) axis alterations

**DOI:** 10.1186/s12888-021-03170-w

**Published:** 2021-03-31

**Authors:** Ana Teresa D. D’Elia, Mario F. Juruena, Bruno M. Coimbra, Marcelo F. Mello, Andrea F. Mello

**Affiliations:** 1 Department of Psychiatry, Federal University of São Paulo (UNIFESP), Rua Major Maragliano, 241, Vila Mariana, São Paulo, SP CEP 04017-030 Brazil; 2 Department of Psychological Medicine, Institute of Psychiatry, Psychology and Neurosciences, Kings College London, London, UK

**Keywords:** PTSD, Neurobiology, Depression, sexual assault, ACTH, HPA axis, Cortisol

## Abstract

**Background:**

Sexual assault is implicated in several adverse psychological and physical health outcomes, including posttraumatic stress disorder (PTSD) and depression. Neurobiological research has shown variations related to the hypothalamic-pituitary-adrenal (HPA) axis, immune alterations, metabolic function, and brain circuitry. Although these mechanisms have been extensively studied, the results have demonstrated different outcomes in PTSD.

**Methods:**

We compared the plasma adrenocorticotropin (ACTH) and salivary cortisol levels of fifty-eight women with PTSD developed after sexual assault to those of forty-four female controls with no history of trauma. We also evaluated the psychiatric diagnosis and symptom severity of PTSD and depression. The participants’ clinical conditions were associated with their hormonal levels to assess whether symptom severity was related to hormonal imbalance.

**Results:**

A large percentage of sexually assaulted women had PTSD and comorbid depression. The ACTH levels were higher in the PTSD group than the control group and increased as PTSD severity increased, considering depressive symptoms, measured by the Beck Depression Inventory (BDI) (*p* < 0.0001), as well as PTSD symptoms, measured by subscale D of the Clinician-Administered PTSD Scale (CAPS-5) (*p* = 0.045) and the CAPS-5 total scale (*p* = 0.026). Cortisol levels measured at 10 pm were higher for the PTSD group than the control group (*p* = 0.045, *p* = 0.037, respectively), and the cortisol awakening response showed elevated cortisol levels for the PTSD group.

**Conclusions:**

These results show a correlation between symptom severity and HPA axis imbalance in patients with PTSD. Elevated ACTH and an elevated cortisol response in patients with comorbid depressive symptoms were the opposite of the expected response for patients with PTSD only. This association leads to the hypothesis that the neurobiological alterations of PTSD are related to the type of symptoms presented and their severity. These manifestations likely influence the disease course, prognosis and response to treatment. These outcomes highlight the need to discuss particular neurobiological alterations in patients with PTSD developed after sexual assault, mainly those with severe depressive symptoms.

**Supplementary Information:**

The online version contains supplementary material available at 10.1186/s12888-021-03170-w.

## Background

Sexual violence against women is a fundamental violation of women’s human rights and a public health problem that affects nearly one-third of women globally [1]. Sexual assault, a less encompassing term used to refer to sexual contact or behaviour that occurs without the victim’s explicit consent, is implicated in several psychological and physical health outcomes [2, 3]. Women victims of sexual assault, including rape, report lower perceived health status; more somatic symptoms; more frequent chronic pain complaints; gynaecological syndromes; and psychiatric disorders such as substance abuse, depression, and posttraumatic stress disorder (PTSD) [4–6].

Rape is one of the most severe types of traumatic events, with a high prevalence of PTSD (up to 50% of victims) as a direct consequence of the trauma [7, 8], and women are three times more likely to experience sexual violence than men. Consistent findings in the literature show a higher conditional general risk in women for the development PTSD, suggesting that conditions linked to gender specificity in neurobiological responses in PTSD can robustly influence the greater risk of developing PTSD in this group [8–10]. Despite the data gathered on the high prevalence of PTSD resulting from this kind of trauma, few studies have addressed the neurobiology of PTSD considering the female population who has experienced sexual violence.

Stress-induced stimulation of the hypothalamic-pituitary-adrenal (HPA) axis is implicated in the activation of the paraventricular nucleus (PVN) on the hypothalamus, which secretes corticotrophin-releasing factor (CRF) into the portal venous circulation that, in turn, stimulates anterior pituitary cells to secrete adrenocorticotropic hormone (ACTH). Increases in circulating ACTH promote glucocorticoid discharge from the adrenal cortex [11].

The HPA axis is a neuroendocrine complex component that directly influences the feedback reactions needed for acute stress adaptation. A model that has been extensively studied in the neurobiology of PTSD shows variations related to the high sensitivity of glucocorticoid receptor (GR) and an increase in the negative feedback inhibition of cortisol in the pituitary, which results in low levels of circulating cortisol [12]. This hormonal imbalance is found in PTSD and a set of characteristic symptoms, such as intrusion symptoms, avoidance, negative alterations in cognition and mood and alterations in arousal and reactivity.

Several types of research have demonstrated different outcomes evidencing diverse biological expressions of PTSD influenced by several conditions, such as the type of trauma, age, gender, early life stress, genetics, and immunological factors. All these aspects may influence the diverse psychopathological expression of PTSD, and variations in biological markers strongly suggest that there are different biological alterations responsible for the development of PTSD symptoms [13].

The presence of depressive symptoms in PTSD patients has been studied frequently due to the high prevalence of comorbidities [14]. The Clinician-Administered PTSD Scale (CAPS-5) is a PTSD severity assessment instrument adapted from the DSM-V criteria, and subscale D of the CAPS-5 aims to assess negative changes in cognition and mood [15, 16]. The symptoms presented in this subscale are sleep alterations (D1), negative beliefs about oneself (D2), guilt (D3), persistent negative emotional state (D4), lack of interest (D5), distance (D6) and inability to feel positive emotions (D7); these symptoms may overlap with depressive symptoms and may signal the presence of this comorbidity in PTSD patients [17–19]. Neurobiology studies on PTSD have shown an unexpected endocrinal imbalance regarding the HPA axis, suggesting particular alterations for PTSD/major depressive disorder (MDD) patients [20–25], but the correlation between prominent symptoms on subscale D and hormonal imbalance has not been analysed.

De Kloet and colleagues demonstrated differences between PTSD patients with and without depression using the dexamethasone-corticotrophin-releasing hormone (CRH) response test. The group with comorbid symptoms showed an attenuated response, with lower ACTH and cortisol responses to the test [20]. Heim et al. showed increased ACTH levels and cortisol in response to a social stressor in women with a history of childhood abuse and MDD [21]. Young and Breslau (2004) demonstrated alterations in cortisol levels, specifically in the evening, in PTSD and MDD groups compared to those in PTSD only and controls [22], and elevated ACTH and cortisol were found in response to CRH infusion in women with PTSD and depressive symptoms [23]. Morris et al. (2012) performed a meta-analysis and verified the differences of HPA axis functioning between three groups, namely, trauma-exposed, PTSD, and PTSD with MDD groups, with the trauma-exposed and PTSD groups showing lower afternoon cortisol levels and the PTSD with MDD group showing higher cortisol levels than the no trauma controls [24]. In addition, a prior study that observed basal cortisol in a trauma-exposed individual with MDD also found a difference between the groups [25].

We reason that HPA functioning and mechanisms should be further studied; the complexity and influencing factors of HPA functioning need to be better understood. The HPA axis has an essential neurobiological role that should be considered to refine and expand neuroendocrine models to identify new possible targets of intervention for severe diseases, which trend to chronicity in severe cases.

In this research, we aimed to investigate ACTH and cortisol levels related to psychopathological aspects in a specific sample of women with PTSD. We hypothesized that the HPA axis response would be blunted, as observed in the literature for PTSD patients, and that this response could change according to the associated depressive comorbid symptoms and history of childhood trauma. Furthermore, we expected to offer new perspectives for the current knowledge of PTSD neurobiology and contribute to broadening the approaches to this disease.

## Methods

### Inclusion and exclusion criteria

The patient group was composed of women aged 18 to 45 who experienced sexual assault up to 6 months before inclusion. To be included, the women had to have a diagnosis of PTSD according to the Diagnostic and Statistical Manual of Mental Disorders (DSM-5) criteria and the CAPS-5, meaning that the trauma occurred at least a month before participation in the study. All patients were referred by the largest public Women’s Health Centre service in São Paulo that offers gynaecological care following sexual assault. The complete diagnostic assessment was based on a structured clinical interview (MINI), and the severity of PTSD symptoms was rated through the CAPS-5 scale.

The controls were voluntarily recruited from the community through advertisements and social media. To be included, the women had to have never been sexually abused (assessed by the CTQ and detailed anamnesis) or had any severe previous or current psychiatric diagnosis (psychosis, bipolar disorder, substance abuse, personality disorder, PTSD or MDD), and they had to be between 18 and 45 years old.

For both groups, the exclusion criteria included severe, chronic, and inflammatory diseases; severe neurological or mental disorders (such as psychosis or bipolar disorder); pregnancy; HIV diagnosis; use of corticosteroids; and treatment with psychotropic medication or psychotherapy. Patients with prior PTSD from other previous traumas were also excluded.

### Participants

Fifty-eight female adult subjects with PTSD resulting from sexual assault and forty-four female controls were recruited from October 2015 to October 2018. The Ethics Committee of the Federal University of São Paulo approved all clinical procedures conducted by the latest Helsinki Protocol. All participants provided written informed consent before participating in this study, which was part of the Thematic Project: Posttraumatic Stress Disorder and Neuroprogression in Women Following Sexual Assault: a Randomized Clinical Trial Evaluating Allostatic Load and Aging Process Acceleration (FAPESP, 2014/12559–5). The details of this study have been published elsewhere [26]. The characteristics of the sample are presented in Table 1.

**Table 1:** Demographic and clinical characteristics

Characteristics	Patients Group *(n = 58)*	Control Group *(n = 44)*	Total *(n = 102)*	*P*	*Effect size*
Age, years					
Mean (SD)	24.6 (6.9)	29.3 (7.5)	26.6 (7.5)	0.002	0.652
Median (Min-Max)	22 (18–45)	27.5 (20–46)	23 (18–46)		
BMI					
Mean (SD)	24.7 (5.11)	24.8 (3.84)	24.7 (4.58)	0.919	0.02
Median (Min-Max)	24 (17.6–41)	23.8 (19.3–35.7)	24 (17.6–24)		
Race^a^, *n (%)*					
White	22 (37.9%)	32 (72.7%)	54 (52.9%)	0.001	0.386
Black	12 (20.7%)	2 (4.5%)	14 (13.7%)		
Mixed (Pardo)	24 (41.4%)	9 (20.5%)	33 (32.4%)		
Other	0 (0%)	1 (2.3%)	1 (1.0%)		
Marital Status *n (%)*					
Single	39 (67.2%)	30 (68.2%)	69 (67.6%)	0.266	0.199
Married	10 (17.2)	10 (22.7%)	20 (19.6%)		
Divorced	2 (3.4%)	3 (6.8%)	5 (4.9%)		
Religion *n (%)*					
Catholic	17 (29.8%)	14 (31.8%)	31 (30.7%)	0.361	0.231
Evangelical	22 (38.6%)	10 (22.7%)	32 (31.7%)		
Spiritualist	1 (1.8%)	3 (6.8%)	4 (4.0%)		
No religion	14 (24.6%)	14 (31.8%)	28 (27.7%)		
Atheist	1 (1.8%)	0 (0.0%)	1 (1.0&)		
Other	2 (3.5%)	3 (6.8%)	5 (5.0%)		
Current Axis I Diagnosis (MINI) *n (%)*					
Depressive Episode	56 (96.5%)	0 (0%)	56 (54.9%)	–	
Suicidal risk	39 (67.2%)	0 (0%)	39 (38.2%)	–	
Depression with melancholic characteristics	43 (74.1%)	0 (0%)	43 (42.1%)	–	

^a^The official race classification in Brazil follows the Brazilian Institute of Geography and Statistics (IBGE) parameters. In this study the racial classification was self-reported

The medical-legal concept of sexual crimes varies from country to country and even between states. We considered rape, attempted rape, forced oral sex, and drug-facilitated rape to be sexual assault events, and in Brazil, these events are legally encompassed in a unique terminology recognized as rape.

### Measures

Two trained researchers from our research group, one psychiatrist and one psychologist, administered all instruments.

#### MINI-international neuropsychiatric interview (MINI)

This brief standard diagnostic structured interview (30 to 45 min long) was developed to identify the criteria for the majority of psychiatric disorders presented in the DSM-IV and ICD-10. Trained psychiatrists interviewed all participants. We used the Brazilian Portuguese version translated and adapted by Amorim et al. [27].

#### Clinician-administered PTSD scale (CAPS-5)

This scale is the most commonly used instrument to assess PTSD diagnostic status and symptom severity in studies. Based on a large-scale psychometric study, the authors of the CAPS stated that there was substantial evidence of both the validity and reliability of the scale as a measure for the symptoms of PTSD; the scale has been adapted to Brazilian Portuguese [16, 28].

#### Beck depression inventory (BDI)

The BDI is a self-report 21-item questionnaire and is used as a clinical measure of depression. The Brazilian Portuguese version was translated and adapted by Cunha [29]. We used the cut-off points proposed by Beck et al. [30] to stratify the severity of symptoms, with the cut off for severe depression being > 30 points. The psychometric properties of the BDI have been reported, with a Cronbach’s alpha of 0.95.

#### Beck anxiety inventory (BAI)

The BAI is self-report 21-item questionnaire that is used as a clinical measure of anxiety. The total score is calculated as the sum of the individual items classifying the severity of anxiety as a minimum, mild, moderate, or severe [31], and those cut-off points were used in our study. The Brazilian Portuguese version was translated and adapted by Cunha [29]. The psychometric properties of the BAI have been reported, with a Cronbach’s alpha of 0.94.

#### Childhood trauma questionnaire (CTQ)

The CTQ is a self-report instrument for adults and adolescents that investigates five early-stage traumatic factors; physical, emotional, and sexual abuse; and physical and emotional negligence. We used the Brazilian Portuguese version, translated and adapted by Grassi-Oliveira et al. [32]. The psychometric properties of the CTQ have been reported, with a Cronbach’s alpha of 0.65 [33].

#### Hormone assays

Salivary cortisol samples were collected with a synthetic swab using the Salivette® kit. All participants were instructed to gently chew on the cotton swab in their homes until it was soaked and then put it back into the provided inner tube and put the tube in the refrigerator. They were instructed to perform this procedure five times: at 10 pm; before going to sleep; and the next day at 6:30 am, 7 am, and 7:30 am. They were requested not to smoke, drink, or eat 2 h before the test, and they were informed that they should also stay in bed until the three morning procedures were completed to minimize possible environmental interference in the results. The night of the day when the participants collected the morning samples, they went to the Sleep Institute at UNIFESP for more exams that were part of the thematic study protocol, and they were instructed to bring the material that was collected and stored according to the instructions. The cortisol detection limit was 0.011 μg/dL.

The second salivary cortisol collection was performed at the Sleep Institute at UNIFESP. A professional collected the participants’ saliva at 10 pm (before the polysomnographic exam) and at 6:30 am, 7 am, and 7:30 am the next morning (after the polysomnographic exam). In addition, one blood sample was collected at 7 am to determine the adrenocorticotropin (ACTH) concentrations using an enzyme-linked immunosorbent assay (ELISA). The ACTH detection limit was 1 pg/mL. To assess the expected increase in morning cortisol levels, we carried out three cortisol measurements in the morning and one at night (when we expected to find lower levels). Thus, it was possible to obtain the cortisol curve and assess whether the functioning of the HPA axis followed the physiological rhythm since early morning cortisol measurements are more reliable. In contrast, measurements at other times appear to be influenced by external stimulation [34, 35].

We followed the protocol for collecting cortisol, setting predetermined times for collection to standardize the data obtained following the guidelines for the assessment of cortisol awakening response to forced awakening [34].

For a more reliable result, the final cortisol measurements were obtained using the mean values of the home and Sleep Institute saliva collections.

### Statistical analyses

The baseline patient characteristics are expressed as the absolute and relative frequencies for categorical variables and as the mean, standard deviation, median, minimum, and maximum for quantitative variables. The associations between categorical variables were evaluated by the chi-squared test or Fisher’s exact test, as appropriate. In addition, we reported Cramer’s V coefficient, and we used an unpaired two-sample *t*-test to test the mean cortisol and ACTH levels for the case and control groups. In addition, we reported the effect size in terms of Cohen’s D. The correlation coefficient between ACTH level and CAPS-5 score was calculated using Spearman’s correlation coefficient.

We fitted a generalized linear model (GLM) to evaluate the relation of the independent variables (age, body mass index (BMI), BDI score, BAI score, and CTQ score) to the ACTH outcome. We assumed a gamma distribution and an identity link function. The variables of the GLM were selected using the Akaike information criterion (AIC), thus yielding the final model. The generalized estimating equation (GEE) was also adjusted to the dataset to evaluate the effects of the covariates, such as time and group, on cortisol outcomes. In addition, we assumed a linear relation between the independent and cortisol variables.

We assumed a gamma distribution, a log link function, and an autoregressive matrix correlation. The significance level was fixed at 5% for all tests. Statistical analyses were performed using IBM SPSS Statistics version 24.0 (IBM Corp., Armonk, NY, USA) and R software version 3.5 (R Foundation for Statistical Computing, Vienna, Austria).

## Results

### Demographics and clinical characteristics

Table 1 presents selected sociodemographic and clinical characteristics of the 58 female PTSD patients and 44 age-matched controls. The mean age of the patient group was 24.6 years, and the participants’ ages ranged from 18 to 45 years old (mean (M) = 22; standard deviation (SD) = 6.8); the mean age of the control group was 24.4 years, and the participants’ ages ranged from 20 to 45 years old (M = 29.0; SD = 0.5; *p* = 0.002). A total of 52.9% of the sample was white (*p =* 0.001*),* and 67.6% was single (*p* = 0.266). All PTSD patients had experienced non-partner sexual assault, even though this was not a prerequisite to participating in the study. The mean time since the index trauma was 51.5 days, the minimum time was 30 days, and the maximum was 157 days (SD = 24.7; median (Md) = 42; first quartile (Q1) = 33; third quartile (Q3) = 61).

### Diagnostic status, PTSD, and depression symptom severity

The results of the diagnostic interview (MINI) indicated that in addition to the PTSD diagnosis, most participants in the patient group, 56 out of 58 (96%), had also had a recent depressive episode. Of these 58 patients, 75% displayed depressive symptoms with melancholic characteristics, and 67% had suicidal ideation, as determined by the MINI. One control presented agoraphobia without panic, one presented dysthymia, and one presented generalized anxiety disorder.

The patients had an average CAPS score of 42, and their scores ranged from 22 to 60 (M = 42.3; SD = 9.2; median (Md) = 41 (22–60)), indicating moderate-severe symptomatology. They also showed severe depressive symptoms on the BDI scale (M = 16.6, SD = 15.3, Md = 12 (0–55)), and when categorized, 46% of them had severe depression. Based on the BAI results, 53% of the PTSD patients had moderate or severe anxiety symptoms (M = 15, SD = 14.6). The controls did not score on either scale.

### ACTH and cortisol levels

There were significantly elevated levels of ACTH in the PTSD patients compared to those in the controls (patients: M = 22.8, SD = 10, Md = 20.5 (7.6–50.3); controls: M = 19, SD = 8.7, Md = 18.4 (4.3–46.8); *p* = 0.045). The mean cortisol level at 10 pm was also significantly elevated in the PTSD group.

**Fig. 1:**
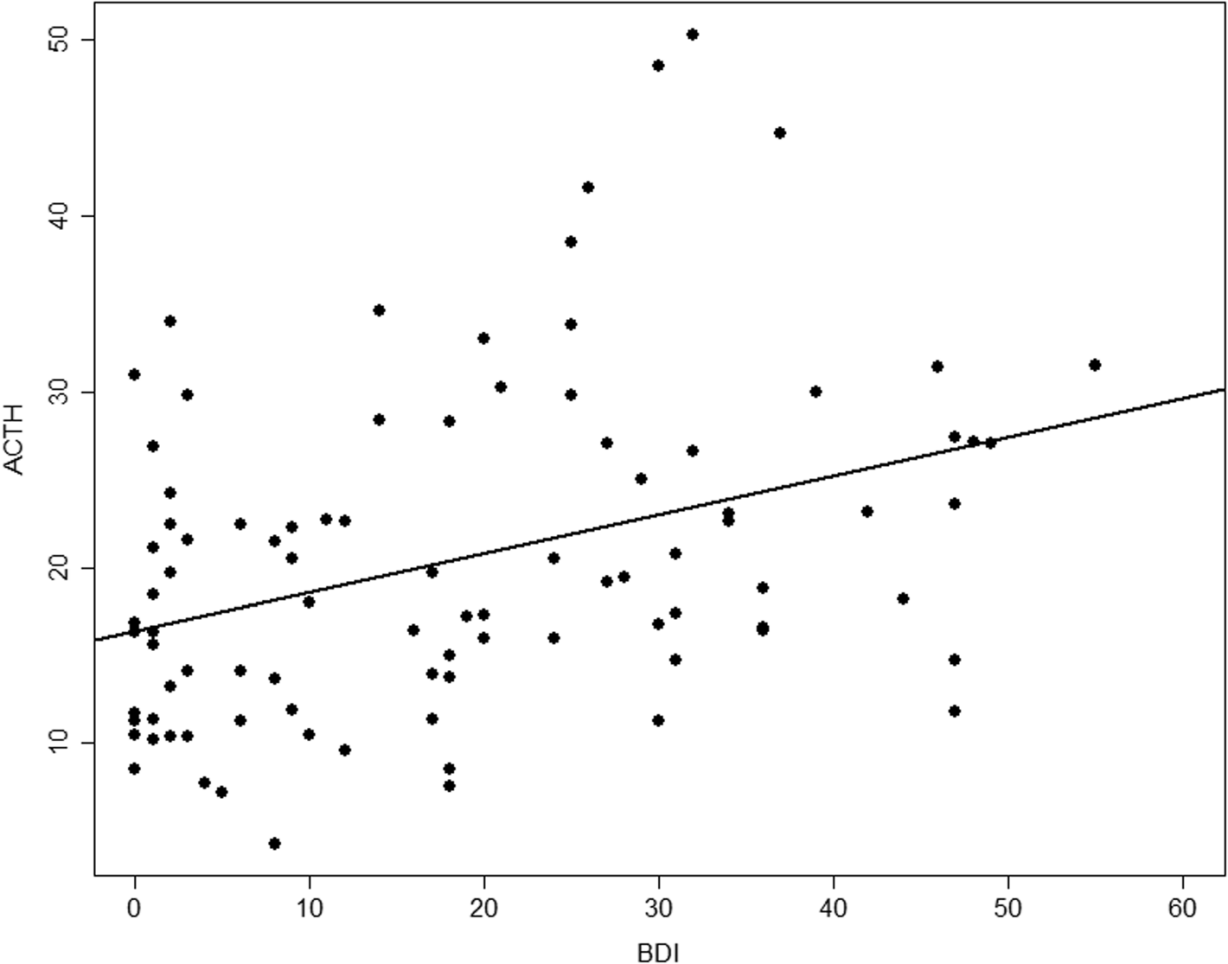
Comparison of ACTH levels considering the severity of depressive symptoms on the sexually abused group with higher ACTH levels as the severity increased on BDI score (*B* = 0.15; *p* < 0.021). It indicates that BDI score is an important factor to the ACTH outcome. In addition, for each unit’s increase on the BDI score, an increase of 0.154 in the ACTH level was expected. Comparison of cortisol levels by time

The GLM considering the variables age, BMI score, BDI score, BAI score, and CTQ score was adjusted to the data to identify the possible factors associated with the ACTH outcome for only the PTSD group. Considering this model, we found that only the BDI score was significant. The final regression model considering only the BDI variable showed higher strength of comparison considering the severity of depressive symptoms. As the severity of the BDI score increased, higher ACTH levels were observed (*B* = 0.15; *p* = 0.021) (Table 2). For each unit increase in the BDI score, an increase of 0.154 in the ACTH level was expected (Fig. 1). The ACTH levels were also related to the CAPS-5 score, demonstrating that increased ACTH levels were related to severe PTSD symptoms (*r* = 0.3, *p* = 0.026).

**Table 2:** Estimates of the parameters of the Generalized linear model with gamma response for ACTH measurement

Complete model					Final model		
Variable	Category	Coefficient	SE	***p***	Coefficient	SE	***p***
Intercept		21.399	6.202	< 0.0001	18.549	1.289	< 0.0001
Group	Case	1.470	5.194	0.778			
AGE	Case	−0.105	0.139	0.450			
IMC	Case	0.011	0.230	0.961			
BDI	Continuous	0.203	0.129	0.121	0.1541	0.066	**0.021**
BAI	Continuous	−4.296	5.336	0.423			
QUESIAF	Continuous	0.335	5.214	0.949			
QUESIAS	Continuous	1.808	3.719	0.628			
QUESINEGLE	Continuous	−1.222	3.687	0.741			

Note: *BMI* Body mass index, *BDI* Beck Depression Inventory, *BAI* Beck Anxiety Inventory, *CTQPA* Childhood Trauma Questionnaire (Physical Abuse Subscale), *CTQSA* Childhood Trauma Questionnaire (Sexual Abuse Subscale), *CTQNEGLE* Childhood Trauma Questionnaire (Neglect Subscale), *SE* Standard Error

**Fig. 2:**
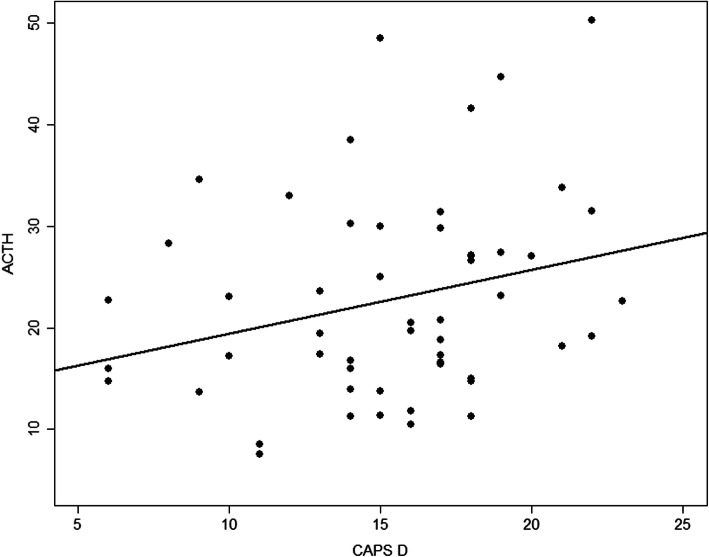
Increase on ACTH levels associated to CAPS-5, subscale D (*r* = 0.227; *p* = 0.045), which refers to negative and mood symptoms connected to PTSD diagnosis, indicating increase on ACTH levels according to the higher scores on the subscale D. Comparison of ACTH levels considering the severity of depressive symptoms on the sexually abused group

After obtaining these results, we analysed data from subscale D of the CAPS-5 to further explore the relationship between negative and depressive symptoms and HPA axis imbalance considering the large number of depressive patients in our sample. We found an association of ACTH levels and higher scores on subscale D of the CAPS-5 (*r* = 0.2; *p* = 0.045), which assesses negative symptoms related to the PTSD diagnosis (Fig. 2).

**Fig. 3:**
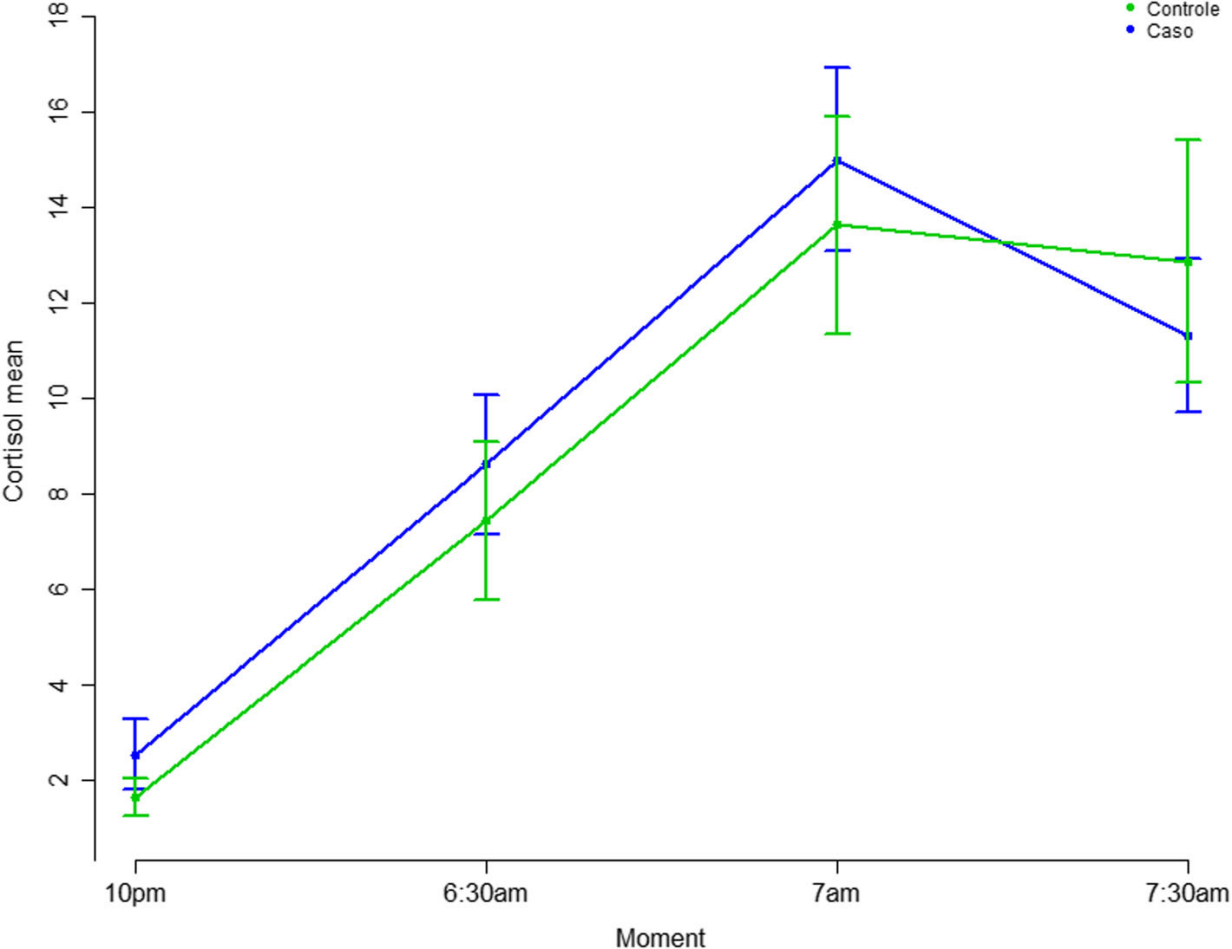
Comparison of cortisol levels by time. At 10 pm the cortisol level was significantly higher in patients (Mp = 2.5, SD = 2.6; Mc = 1.6, SD = 1.2; *p* = 0.037). There are higher general cortisol levels demonstrating increased HPA axis response in the PTSD group. The ACTH levels associated to CAPS-5, subscale D

The comparison between groups using the *t*-test showed increased cortisol levels for the PTSD group and a significant increase at 10 pm (patients: M = 2.5, SD = 2.6, Md = 1.5(0.3–13.6); controls: M = 1.65, SD = 1.2, Md = 1.3 (0.3–5.4); *p* = 0.037) (Fig. 3). The generalized estimation regression model was adjusted considering the group (case and control) and time (10 pm, 6:30 am, 7 am, and 7:30 am) variables and demonstrated an effect of both (*p* = 0.021 and *p* = < 0.0001, respectively). The results showed a significant interaction effect of group × time, with elevated cortisol levels for the PTSD group, which showed higher cortisol levels, on average (Table 3). The BMI, BAI and CTQ scores were removed from this model since correlations were not confirmed in previous analyses. There was no significant difference between the cortisol areas under the curve (AUC) collected at home and in the laboratory (AUC-home: M = 54.8, SD = 41; AUC-laboratory: M = 57.9, SD = 36, *p* = 0.575). When the BDI score and the score of subscale D of the CAPS-5 were added to the model, the results were not significant. The time from the onset of trauma until the inclusion of patients in the study did not influence the ACTH levels (β = − 0.002, SE = 0.003, *p* = 0.539) or the AUC of cortisol (β = − 0.004, SE = 0.002, *p* = 0.089).

**Table 3:** Estimates of the parameters of the GEE model with gamma response for cortisol measurements

Parameter	***Coefficient***	Standard Error (SE)	CI 95% Wald		***p***
			Lower	Upper	
Intercept	0.504	0.116	0.277	0.731	**< 0.0001**
PTSD Group	0.426	0.184	0.065	0.788	**0.021**
Control Group	Ref				
7:30 am	2.051	0.131	1.795	2.307	**< 0.0001**
7 am	2.108	0.114	1.884	2.331	**< 0.0001**
6:30 am	1.502	0.123	1.262	1.743	**< 0.0001**
10 pm	Ref				
PTSD*7:30 am	−0.555	0.189	−0.926	−0.185	**0.003**
PTSD*7 am	−0.331	0.179	−0.682	0.021	0.065
PTSD*6:30 am	−0.279	0.182	−0.636	0.077	0.124
Scale	0.458				

Only two women had a diagnosis of PTSD without concomitant MDD; thus, it was impossible to carry out a comparative analysis between women with PTSD and MDD and women with PTSD without MDD.

## Discussion

Despite the intrinsic complexity of the neurobiology of PTSD and the multiple factors that may influence it, this study shows a relationship between increased levels of ACTH and the severity of PTSD symptoms, mainly depressive symptoms, in a sample of women recently exposed sexual assault.

An extensive review by Zoladz and colleagues [13] revealed several conflicting results concerning neuroendocrine alterations and PTSD, and the authors reported that fluctuations in HPA axis hormone levels in PTSD were induced by several factors, such as gender, childhood trauma, MDD, and life habits, such as smoking or physical activity. Considering the diverse neuroendocrine alterations that have been associated with these variables in the PTSD literature, we suggest that PTSD may not be considered a single-faceted disorder and may not be characterized by a specific biological alteration. This leads us to the hypothesis that this diversity of biological alterations may be related to how PTSD presents itself, including severity symptoms and comorbidities, such as MDD, which was observed in our sample.

Comorbid conditions are expected in PTSD, and according to one meta-analysis, MDD is present in almost 50% of cases [36]. Several studies have reported that concomitancy between PTSD and another psychiatric disorder (especially MDD) may be as high as 80% [37]. Since the PTSD diagnosis criteria have changed over the years and the DSM-5 proposes cluster D symptoms (Negative Alterations in Cognitions and Mood), a question has been raised regarding whether depression should be considered a comorbidity added to PTSD or considered part of PTSD. Although the diagnoses of MDD are broader and include other symptoms, subscale D identifies signs of emotional blunting, anhedonia and distorted cognition, which may represent an overlap of symptoms. The presence of those negative mood symptoms could be considered a specific phenotype of PTSD with more significant cognitive impairment [38], increased suicidal levels [39], and even distinguished biological alterations compared to those in PTSD without significant depressive symptomatology [14].

In our sample, there was a high prevalence of PTSD with a depressive episode according to the MINI. We also found that 46% of the sample had severe depressive symptoms according to the BDI; moreover, based on the CAPS-5 subscale D, all patients presented negative cognition and mood symptoms. These results show the correlation of depressive symptoms and the increased HPA axis response in this sample. The increase in ACTH levels was related to depressive symptoms and the severity of PTSD symptoms, particularly related to the subscale of the CAPS-5, which also refers to negative mood alterations (subscale D). This characteristic of our sample led us to an important limitation: we could not compare patients with and without MDD, and the hypothesis regarding the hypo-responsiveness of the HPA axis was not supported.

Depression pathophysiology associated with PTSD may have influenced these results. In depression disorders, it is common to find impaired HPA axis negative feedback, with elevated CRF, an increased number of ACTH secretory episodes, and high plasma cortisol levels [40], which is the opposite HPA axis biomarker of PTSD with a profile of blunted cortisol and ACTH levels [7]. We speculate that these negative symptoms, identified by the MINI as a depression diagnosis, are part of a PTSD subgroup with different biological alterations from those observed in PTSD only, stimulating the HPA axis to respond similarly as in depression.

This study showed higher general cortisol levels in the patient group than the control group; perhaps the presence of depressive symptoms influences HPA axis functioning in a pattern that is more similar to the biological alterations shown in depression [40–42]. There may be a difference in the HPA axis response pattern of individuals with PTSD only and PTSD associated with depressive symptoms. Yehuda et al. demonstrated a difference between a PTSD with no MDD group and a PTSD with MDD group regarding GR gene epigenetic methylation, showing lower methylation in the PTSD group with no MDD than in the PTSD group with MDD [43]. Interestingly, the lower rates of methylation correlate with the increase in GR sensitivity, which implies an increase in negative feedback and suppression of the HPA axis, which, in turn, is related to PTSD physiopathology. According to our results, the GR may not be very sensitized, which could be why the HPA axis is not blunted, as is common in PTSD. Perhaps the genetic difference in GR functioning may indicate some vulnerability to depressive symptoms in PTSD. This could generate an alternative HPA axis response that does not correspond to any of the two isolated diseases, but it is more similar to the depression pathway than PTSD only.

The increase in evening cortisol has already been reported in several previous studies, consistent with our findings that indicated increased saliva and urinary cortisol in women with PTSD and depression [22, 44]. Later analyses conducted with a larger sample showed an increase in evening cortisol in PTSD and MDD [22]. It is likely that depressive symptoms influence HPA axis functioning, altering the physiological circadian rhythm in a rather specific manner, such as the increased 10 pm cortisol levels in PTSD patients compared to those in controls at the same time in this study. Consistent with this hypothesis, Morris et al.’s meta-analysis showed a difference between the groups of patients with PTSD and those with PTSD with MDD, with afternoon cortisol levels being increased in patients with PTSD with MDD [24]. These findings suggest the need to define a different biological function for these patients (with PTSD and current MDD) with singular alterations in the HPA axis, which likely influence other response mechanisms to stress, such as inflammation and immunity.

It is warranted to analyse these results from the perspective of PTSD chronicity. Previous evidence showed that PTSD chronicity could lead to increased inflammatory markers and oxidative stress. The maintenance of HPA axis activation for extended periods may be related to deleterious consequences due to the effects of cortisol toxicity in brain areas, e.g., the hippocampus and the prefrontal cortex [45, 46]. Our findings with recently assaulted women that related the severity of PTSD symptoms to changes in the HPA axis at the onset of PTSD might differ from those for chronic PTSD, when more serious biological changes are expected. Studies investigating how these early biological changes interfere with late manifestations and the effects of early treatment on these alterations are essential to elucidate PTSD pathophysiology. Another limitation of our study is that we did not collect ACTH and cortisol levels monthly after the participants’ inclusion. We believe that for an in-depth analysis of hormonal imbalance and chronicity, the ideal scenario would be to have a series of evaluations and to reassess the patients after at least 1 year.

These results shed light on the need for future studies to evaluate how depressive symptoms in PTSD influence the neurobiological response and whether it generates a different functioning of the HPA axis in PTSD patients. HPA axis functioning in PTSD is very complex and can be influenced by diverse symptoms. Additionally, it is important to note that the impact of sexual assault may be so profound that the severity of symptoms we observed is more the rule than the exception in these cases and may shape a specific kind of biological response, which requires further understanding. Another factor that can affect PTSD neurobiology and that should be highlighted—especially in our sample—is gender differences in biological responses. In this sense, we should consider the possible influence of these factors on the outcome. Similar to the present study findings, a recent review showed that ACTH variations by gender are mostly related to neurobiological factors connected to gender differences since women with PTSD presented increased ACTH levels, while men presented blunted levels [47]. Likewise, Groer et al. showed increased ACTH levels in women who were raped one to 3 days after the trauma, but cortisol levels did not correspond to the ACTH increase [48].

We acknowledge several additional limitations of this study. First, women were referred from another hospital, and we have to consider that PTSD patients usually present high dropout rates [49], which impacted the need for a quick appointment. In this sense, it was impossible to standardize blood collection at the same menstrual cycle phase. The role of female hormones in PTSD physiopathology has been studied as an aspect that can clarify this female PTSD profile. The effects of gonadal steroids on elements of the HPA axis in females may influence the increased ACTH levels in response to acute and chronic stressors [50, 51].

Another point to be considered is that some of the women were using oral contraceptives, which can influence the HPA axis response to trauma. It is also worth stressing that it is impossible to guarantee that the saliva collection conducted at the participants’ homes was conducted correctly, even though the participants were given instruction. There might have been a difference between early measurements in the first month after the event compared to those taken 6 months later. Few studies of PTSD in sexually assaulted women have been conducted; however, more studies with larger samples are needed to present more comprehensive results.

It is essential to note that the cortisol collection method is still controversial in the literature [52]. Even though plasma/serum assays can determine the cortisol level, there are limitations to this method, including differences in affinity and cortisol antibody specificities. Although a saliva sample can be collected at home, at-home collection is often criticized since the technique may involve interference, for instance, related to eating habits and possible contamination of the sample. The literature does not provide a consensus as to the best method of cortisol collection. We opted for saliva collection since it can be carried out at home and is less invasive. Saliva was also collected at the lab to minimize bias, so we had two cortisol measurements, which provided us with a more reliable result. Having a single ACTH dose is still a limiting factor since this secretion is also pulsatile and maintains the circadian rhythm.

## Conclusions

The alterations observed in our study are similar to other recent literature results supporting the hypothesis that the biological response in PTSD may be variable depending on comorbid symptoms and their severity. The presence of depressive symptoms in PTSD patients may be related to HPA axis imbalance and increases in ACTH and cortisol levels, which can be related to a series of events, such as inflammation, accelerated ageing and chronicity of diseases. More studies are needed to enhance the understanding of the neurobiological bases of PTSD and offer new perspectives regarding its treatment and prognosis.

## Supplementary Information

**Additional file 1.**

## Data Availability

The datasets used during the current study are available from the corresponding author on reasonable request.

## Abbreviations

AIC: Akaike Information Criterion
AUC: Area Under the Curve
ACTH: Adrenocorticotrophic hormone
BAI: Beck Anxiety Inventory
BDI: Beck Depression Inventory
BMI: Body mass index
CAPS-5: Clinician-Administered PTSD Scale
CRF: Corticotrophin-releasing factor
CRH: Corticotrophin-releasing hormone
CTQ: Childhood Trauma Questionnaire
DSM-5: Diagnostic and statistical manual of mental disorders
ELISA: Enzyme-Linked-Immuno-Sorbent Assay
GEE: Generalized estimating equation
GLM: Generalized linear model (GLM)
GR: Glucocorticoid receptor
HPA: Hypothalamic-pituitary-adrenal
M: Mean
Md: Median
MDD: Major Depressive Disorder
MINI: Mini-International Neuropsychiatric Interview
PTSD: Posttraumatic stress disorder
PVN: Paraventricular nucleus
SD: Standard deviation
SE: Standard Error
