# Supplementary material for: Posttraumatic stress disorder (PTSD) and depression severity in sexually assaulted women: hypothalamic-pituitary-adrenal (HPA) axis alterations
Source: BMC Psychiatry. 2021 Mar 31;21:174. doi: 10.1186/s12888-021-03170-w (PMC8010966; doi:10.1186/s12888-021-03170-w)
Supplement: Supplementary file 1 — Additional file 1. [file 12888_2021_3170_MOESM1_ESM.pdf]

Identification sheet and questionnaire socio-demographic

Name:
Date:
Birth date:
Age:
Place of birth:
Address:
Email:

Weight:
Height:
Blood pressure:
Abdominal circumference:
Pulse:

Questionnaire socio-demographic

Your racial identity:
- 1-White
- 2-Black (Afro-Brazilian)
- 3-Asian
- 4-Pardo (Mixed)
- 5-Indigenous
- 6-Other

Your marital status:
- 1-Single
- 2-Separated
- 3-Divorced
- 4-Married
- 5-Widow
- 6-Cohabiting

Your religion:
- 1-Catholic
- 2-Evangelical
- 3-Spiritualist
- 4-No religion
- 5-Atheist
- 6-Other

Your education level:
- 1-Illiterate
- 2-Incomplete first degree
- 3-Complete first degree
- 4-Incomplete high school
- 5-Complete high school
- 6-Incomplete university
- 7-Complete University

8-Incomplete graduate
9-Complete graduate

Complete years of study:
Your profession:
Your employment status:
1-Employment
2-Unemployment

Your individual mensal income (R$):
What is the total number of people living with you?
What is the total income of all the people who live with you? Except you.
What is the total number of rooms in your home?

* *The official race classification in Brazil follows the Brazilian Institute of Geography and Statistics (IBGE) parameters. The socio demographic questionnaire was self-reported.*
